# Projected Future Distributions of Vectors of *Trypanosoma cruzi* in North America under Climate Change Scenarios

**DOI:** 10.1371/journal.pntd.0002818

**Published:** 2014-05-15

**Authors:** Miroslava Garza, Teresa Patricia Feria Arroyo, Edgar A. Casillas, Victor Sanchez-Cordero, Chissa-Louise Rivaldi, Sahotra Sarkar

**Affiliations:** 1 Department of Biology, The University of Texas–Pan American, Edinburg, Texas, United States of America; 2 Laboratorio de Sistemas de Información Geográfica, Instituto de Biología, Universidad Nacional Autónoma de México, Distrito Federal, Mexico; 3 Section of Integrative Biology, University of Texas at Austin, Austin, Texas, United States of America; Universidad Autónoma de Yucatán, Mexico

## Abstract

**Background:**

Chagas disease kills approximately 45 thousand people annually and affects 10 million people in Latin America and the southern United States. The parasite that causes the disease, *Trypanosoma cruzi*, can be transmitted by insects of the family Reduviidae, subfamily Triatominae. Any study that attempts to evaluate risk for Chagas disease must focus on the ecology and biogeography of these vectors. Expected distributional shifts of vector species due to climate change are likely to alter spatial patterns of risk of Chagas disease, presumably through northward expansion of high risk areas in North America.

**Methodology/Principal Findings:**

We forecast the future (2050) distributions in North America of *Triatoma gerstaeckeri* and *T. sanguisuga*, two of the most common triatomine species and important vectors of *Trypanosoma cruzi* in the southern United States. Our aim was to analyze how climate change might affect the future shift of Chagas disease in North America using a maximum entropy algorithm to predict changes in suitable habitat based on vector occurrence points and predictive environmental variables. Projections based on three different general circulation models (CCCMA, CSIRO, and HADCM3) and two IPCC scenarios (A2 and B2) were analyzed. Twenty models were developed for each case and evaluated via cross-validation. The final model averages result from all twenty of these models. All models had AUC >0.90, which indicates that the models are robust. Our results predict a potential northern shift in the distribution of *T. gerstaeckeri* and a northern and southern distributional shift of *T. sanguisuga* from its current range due to climate change.

**Conclusions/Significance:**

The results of this study provide baseline information for monitoring the northward shift of potential risk from Chagas disease in the face of climate change.

## Author Summary

Chagas disease kills thousands of people annually. Triatomine insects (family Reduviidae, sub-family Triatominae), can be potential vectors of the parasite (*Trypanosoma cruzi*) that causes the disease. There are often no symptoms until cardiac and digestive system dysfunction (possibly including heart failure) after 10 to 30 years of infection. Climate change can shift the distribution of triatomine insects, favoring the spread of the disease to non-original areas. We used distributional information on the most commonly found triatomine species and the most important vectors of *Trypanosoma cruzi* in South Texas and North Mexico (*T. gerstaeckeri* and *T. sanguisuga*), and explanatory climatic variables to forecast the potential distribution of the insects in the year 2050. We used two different scenarios of climate change and three different general circulation models. Our results showed that the triatomine species studied will likely shift their distribution northwards in the future. There is thus a need to monitor areas that are not currently endemic for Chagas disease but may potentially be affected in the future due to climate change.

## Introduction

Climate change has been implicated in shifts of the geographic distribution of many species[1], enabling some taxa to increase their distributions into northern latitudes [1], [2]. Thus, changes in climate can potentially alter the spatial range of vector-borne diseases through shifts in geographical distributions of their vectors [3], [4], [5]. Despite some positive developments such as better access to clean drinking water, lower exposure to insect vectors, and higher-quality housing, the projected changes in climate over the next decades may exacerbate infectious disease incidence even in developed regions such as North America [6]. Habitat changes, alterations in water storage and irrigation habits, pollution, development of insecticide and drug resistance, globalization, tourism and travel are additional factors that may help to aggravate this threat [4].

The southern United States is highly vulnerable to outbreaks of vector-borne diseases due to many factors, including poor housing conditions, suboptimal drainage, lack of electricity in some areas, the presence of feral dogs, and human migration [7], [8], [9]. Moreover, that some southern states, such as Texas, share a legacy of neglected tropical diseases (NTDs [9]) with Mexico, increases the urgency of the development and deployment of active surveillance programs necessary for optimal management and control of vector-borne diseases including Chagas disease [7], [9] and leishmaniasis [5].

Chagas disease is a zoonosis caused by *Trypanosoma cruzi*, a flagellated protozoan parasite. *Trypanosoma cruzi* is transferred from mammalian reservoirs (e.g., *Neotoma* woodrats) to humans through a triatomine vector [7]. These vectors are insects from the family Reduviidae, sub-family Triatominae [7], [10]. *Trypanosoma cruzi* is most characteristically transmitted by infected feces of triatomines entering the human bloodstream. However, it can also be transmitted through blood transfusion, organ transplants and ingestion of infected food; congenital parasite transmission has also been demonstrated [7]. After contamination with the parasite, Chagas disease develops from an acute phase (period during which the parasites can be found easily in the blood) followed by an asymptomatic period of varying length; this stage is called the indeterminate phase. During the indeterminate phase, the parasites disappear from the blood. A chronic phase can be followed after 5 to 40 years, and ∼30% of infected people develop the disease [11], [12].

Chagas disease kills approximately 45,000 people annually [13] and affects 10 million people in several countries of Latin America [14]. In the United States around 300,000 individuals could be infected with *T. cruzi*, causing a considerable disease burden [15]. Several factors might influence the geographical distribution of *Trypanosoma cruzi* vectors and reservoirs (e.g., historical presence, the existence of barriers and dispersal capabilities), but anthropogenic factors play a fundamental role in the spread of the disease (e.g., through habitat changes, globalization, and travel [4]). The geographical distribution of Chagas disease has increased beyond regions of endemic occurrence during the last half-century and is now considered a worldwide problem [10].

Species distribution models (SDMs) based on machine-learning algorithms and Geographic Information Systems (GIS) platforms have been used to predict areas of potential distribution of *Trypanosoma cruzi* vectors [7], [16], [17], [18], [19]. These analyses typically show that climatic factors significantly influence the potential geographic distributions of vector (and reservoir) species. Additionally, temperature may have a particularly strong influence on the behavior of triatomine species [20], [21]. For instance, temperatures exceeding 30°C combined with low humidity,cause insects toincrease their feeding rate to avoid dehydration. In addition, in domestic life cycles, when indoor temperatures increase, the insects may develop shorter life cycles and higher population densities [20]. High temperatures can also speed up the development of *T. cruzi* in vectors [22].

In this paper, we forecast the future (2050) distribution in North America of *Triatoma gerstaeckeri* and *T. sanguisuga*, two of the most commonly found triatomine species and important vectors in the southern United States [7]. *Triatoma gerstaeckeri* is one of the most widely distributed *Triatoma* species in Texas [7], occurring mainly in the southern areas of the state. It is also found in New Mexico and in northeast Mexico [7]. *Triatoma gerstaeckeri* is more frequently found in economically poorly-developed areas; though it is naturally found in sylvan environments, it is able to disperse to human dwellings [23]. *Triatoma sanguisuga* can be found in several environments similar to *T. gerstaeckeri*, including domestic surroundings [24]. *Triatoma sanguisuga* has been found in several states across United States including Alabama, Arizona, Florida, Georgia, Kansas, Kentucky, Louisiana, Maryland, Mississippi, Missouri, New Jersey, New Mexico, North Carolina, Ohio, Oklahoma, Pennsylvania, South Carolina, Tennessee, Texas, and Virginia [24]. The species has also been found near the Canadian border in Illinois and Indiana [20]. We used geographic information (longitude/latitude distributional data) (Tables S1 and S2) and explanatory climatic variables (temperature, precipitation, etc., Table 1) to produce Species Distribution Models (SDMs) using a maximum entropy algorithm. Current SDMs were projected to 2050 using three different Global General Circulation models (the Canadian Centre for Climate Modelling and Analysis (CCCMA), the Commonwealth Scientific and Industrial Research Organization (CSIRO) and the Hadley Centre for Climate Change (HADCM3). We used two scenarios A2A and B2A from the International Panel on Climate Change [1]. Our aim was to analyze how climate change might affect the future spread of Chagas disease in North America.

**Table 1:** Bioclimatic variables used to model present and future distribution of *Triatoma gerstaeckeri* and *T. sanguisuga*.

Variable	Explanation
BIO1	Annual Mean Temperature
BIO2	Mean Diurnal Range (Mean of monthly (max temp - min temp))
BIO3	Isothermality (P2/P7) (* 100)
BIO4	Temperature Seasonality (standard deviation *100)
BIO5	Max Temperature of Warmest Month
BIO6	Min Temperature of Coldest Month
BIO7	Temperature Annual Range (P5–P6)
BIO12	Annual Precipitation
BIO13	Precipitation of Wettest Month
BIO14	Precipitation of Driest Month
BIO15	Precipitation Seasonality (Coefficient of Variation)
BIO16	Precipitation of Wettest Quarter
BIO17	Precipitation of Driest Quarter
BIO18	Precipitation of Warmest Quarter
BIO19	Precipitation of Coldest Quarter

Temperature is measured in °C. Precipitation is measured in mm.

## Materials and Methods

### Geographic data

For modeling purposes, geographic data (i.e., longitude and latitude) were gathered from data bases from museum collections, voluntary collectors, and through field work by members of our team in South Texas. For the original field work reported here, insects were collected either from public lands or donated by the owners of private lands. As a pilot study, field work was conducted in one sylvatic area, “La Sal del Rey”, Texas (26° 31′ N and 98° 03′ W), on 8 July 2011. We did not collect insects in domestic areas, we only included the La Sal del Rey locality in the model construction. To collect the insects, we used suspended dark ultraviolet light traps with a white background sheet and baited with carbon dioxide from dry ice. All geographic localities for both species are reported in Supplemental files (Tables S1 and S2). Following the methodology of Sarkar et al. [7], only post-1980 records with an estimated error of <1.0 km were used; these choices ensured compatibility between the resolution of the occurrence data and the spatial and temporal resolution of the environmental layers.

### Study area

The study area includes the continental portions of Mexico and the United States and was delimited in the south by the 14°55′S line of latitude and to the north by the 49° 38′N line of latitude, continued by the lines −66° 97′E boundary and −124° 71′W. It was divided into 14 520 497 cells with an average area of 1.03 km^2^ (SD  = 0.27). This ensured the enclosure of all points used in the analysis.

### Model building and evaluation

Present and projected future potential distributions for the target species were computed using presence records for the species (longitude/latitude) and with climatic parameters as exploratory variables, using a maximum entropy algorithm incorporated in the Maxent software package [11], [25]. Maxent predicts probability values (thresholds) from 0 (least suitable) to 1 (most suitable) of habitat suitability over the study area [11], [25]. We used Maxent Version 3.3.3k (http://www.cs.princeton.edu/~schapire/maxent/) with the default modeling parameters (convergence threshold  = 10^5^, maximum iterations  = 500, regularization value β =  auto) [26]. Climatic variables were selected from the 19 WorldClim variables [27] available at WorldClim database. Following Sarkar et al. [7], four climatic variables were eliminated from the analysis since these variables have presumed artifactual discontinuities for Texas (mean temperatures of the wettest quarter, driest quarter, warmest quarter, and coldest quarter; Table 1). These climatic variables have a resolution of approximately 1×1 km^2^ (more accurately, 30 arc-seconds). Twenty models were developed and evaluated via cross-validation per species. The final model presented is the average of the replicates. Model results were processed and visualized using ArcGIS 10.

For the future climate projections we used three GCMs: the Canadian Centre for Climate Modelling and Analysis (CCCMA), the Commonwealth Scientific and Industrial Research Organization (CSIRO) and the Hadley Centre for Climate Change (HADCM3). We used two scenarios of climate change, A2A and B2A, from the International Panel on Climate Change (IPCC 2007). Both scenarios assume a more heterogeneous world and are oriented toward regionalization. The A2A scenario assumes an increase in population, economic development, regionally oriented and per capita economic growth and technological change that is more fragmented than the scenario B2A. The focus of this scenario is more economic. On the other hand, the B2A scenario describes a world in which the emphasis is on local solutions to economic, social and environmental sustainability. It assumes a constant increase of population, but at a rate lower than A2A and intermediate levels of economic development as well. This scenario is oriented towards environmental protection and social equity.

### Model evaluation

We calculated the Area Under the Curve (AUC) of Receiver Operating Characteristic plots (ROC); [28] to evaluate the models by cross-validation of the 20 replicates using the training and test data as described above. Receiver Operating Characteristic is a threshold–independent measure that evaluates the sensitivity (probability that the model produces a positive result in a positive locality) versus the specificity (probability that the model produces a negative result in a negative locality) of a model when presented with new data. A ROC plot is obtained by plotting the sensitivity on the *y*–axis versus one minus specificity for all available decision thresholds on the *x*–axis. The theoretically perfect result is AUC  = 1, whereas a test performing no better than random yields AUC  = 0.5. The AUC was calculated internally by Maxent. The final AUC is the average AUC for all maps.

### Shifts in suitable habitat in the future

The averaged habitat suitability spatial distributions were converted into binary maps for further analysis using two thresholds: a “minimum training presence threshold” and a 0.5 habitat suitability threshold. A “minimum training presence threshold” is a threshold in which at least one known presence for the target species was found; therefore it guarantees that all presences are predicted as suitable [29]. Shifts on suitable habitat were calculated in km^2^. Percentage of change in suitable habitat comparing present and future projections was calculated using the formula ((future gain - future loss)*100)/present area.

## Results

**Figure 1:**
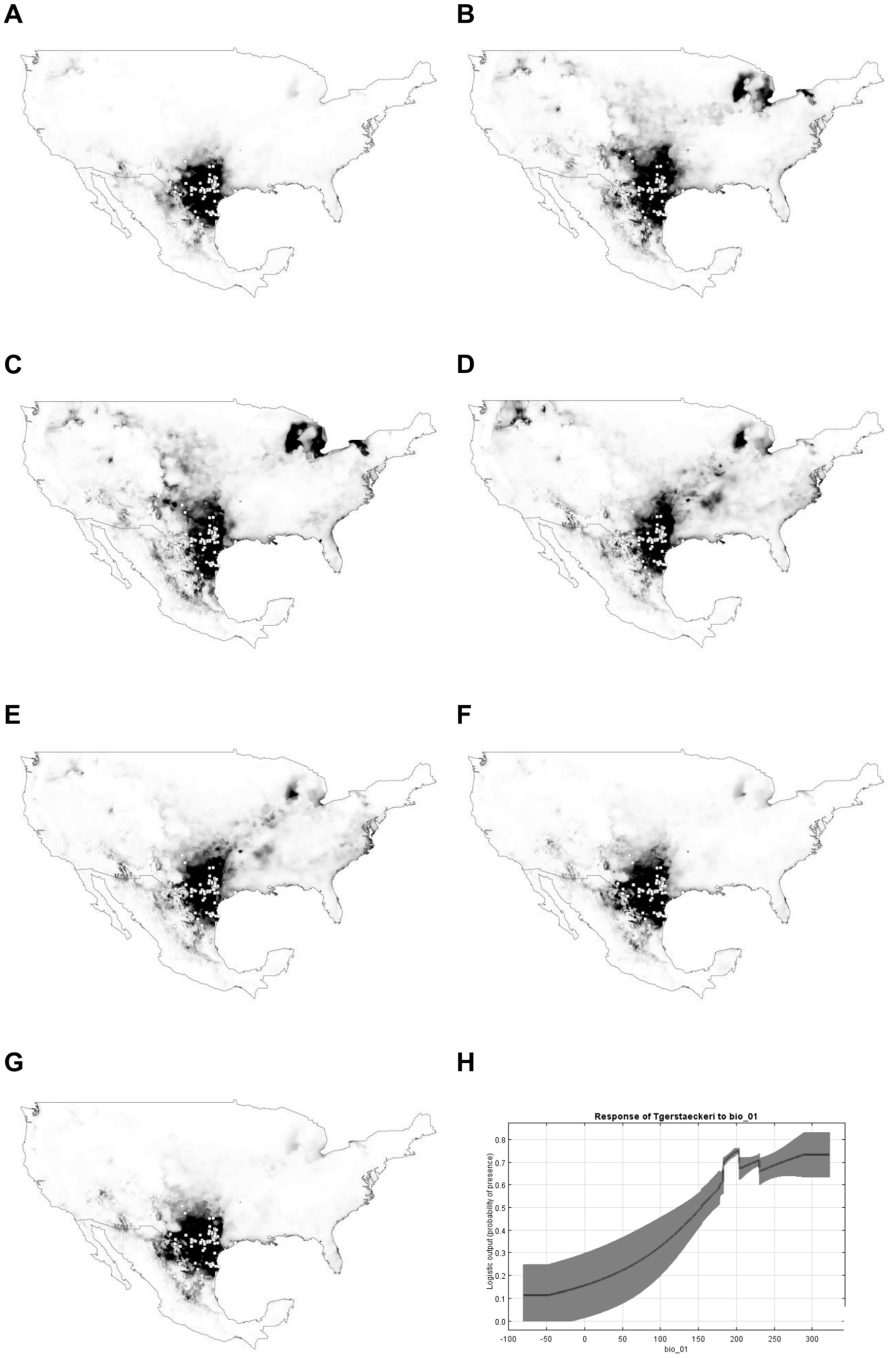
Present (A) and future (2050; B–G) potential distribution for *Triatoma gerstaeckeri*. All models predict a shift in the distribution of this species towards northern and eastern regions of Mexico and USA. Black color =  high suitable habitat *vs*. white color =  no suitable habitat for the species. General circulation models and climatic scenarios: B =  CCCMA-A2A; C =  CCCMA-B2A; D = CSIRO-A2A; E = CSIRO-B2A; F = HADCM3_A2A; G = HADCM3_B2A. Variable with most contribution on the species distribution was Annual Mean Temperature (H), which as per the original data (www.worldclim.org) was multiplied by 10.

**Figure 2:**
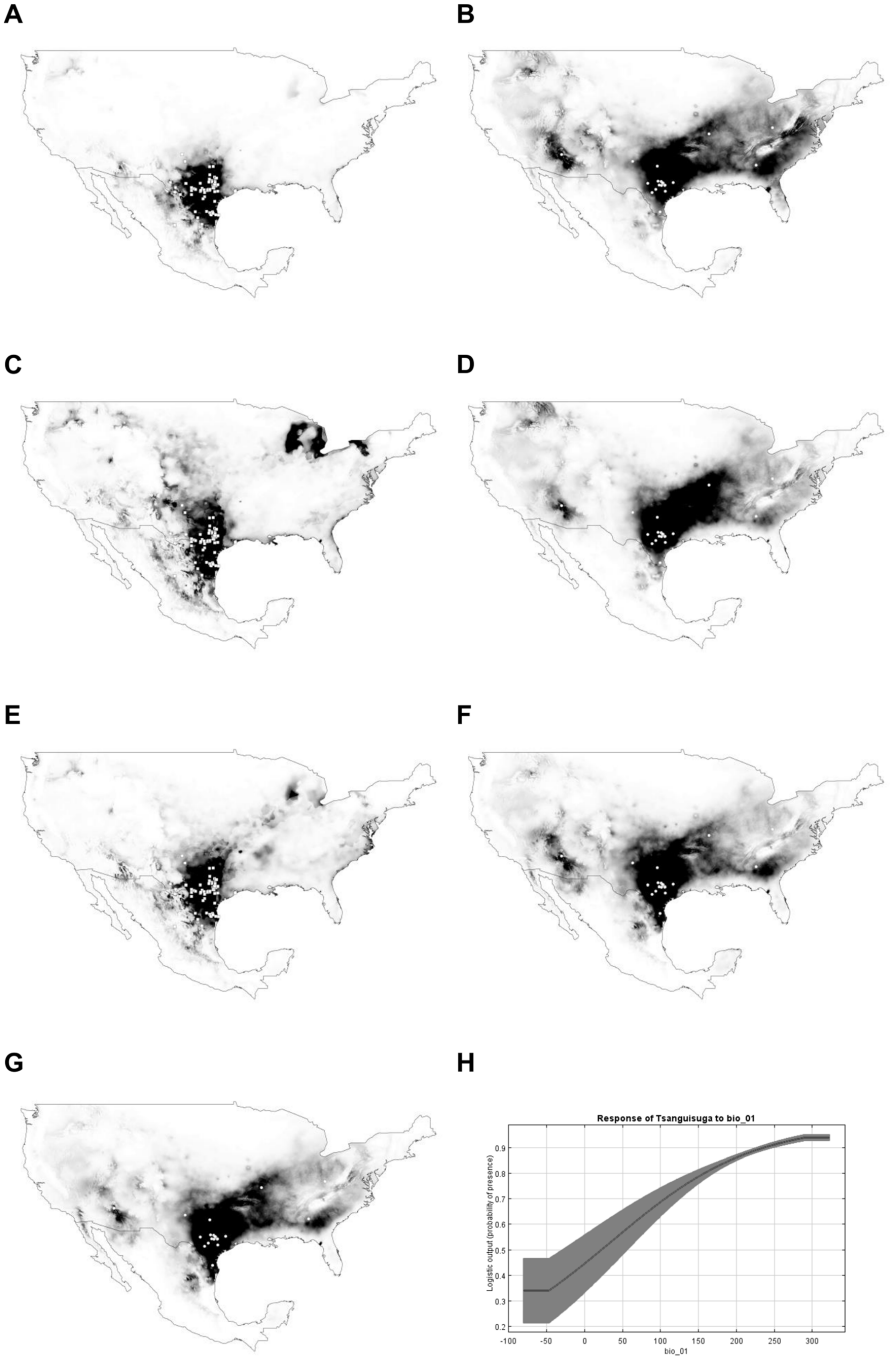
Present (A) and future (2050; B–G) potential distribution for *Triatoma sanguisuga*. All models predict a shift in the distribution of this species towards northern and eastern regions of Mexico and USA. Black color =  high suitable habitat *vs*. white color =  no suitable habitat for the species. General circulation models and climatic scenarios: B =  CCCMA-A2A; C =  CCCMA-B2A; D = CSIRO-A2A; E = CSIRO-B2A; F = HADCM3_A2A; G = HADCM3_B2A. Variable with most contribution on the species distribution was Annual Mean Temperature (H), which as per the original data (www.worldclim.org) was multiplied by 10.

A total of 84 unique geo-referenced localities, i.e., one locality per cell, were used to develop models of present and future suitable habitat for *Triatoma gerstaeckeri* and 24 for *T. sanguisuga* (Tables S1 and S2). Table 2 shows AUC values. For *T. gertaeckeri* the averages AUC were 0.9857 (SD = 0.0015) and 0.9738 (SD = 0.0279) for training and testing data, respectively; for *T. sanguisuga* the corresponding numbers were 0.9680 (SD = 0.0026) and 0.9323 (SD = 0.0982). Figures 1 and 2 show models of present and future distributions for both species.

**Table 2:** Area Under the Curve (AUC) values after MaxEnt.

		Mean	St. Dev	Maximum	Minimum
*Triatoma gerstaeckeri*	Training	0.9857	0.0015	0.9880	0.9826
	Test	0.9738	0.0279	0.9970	0.8935
*T. sanguisuga*	Training	0.9680	0.0026	0.9748	0.9648
	Test	0.9323	0.0982	1.00	0.6912

Models of future distribution for the suitable habitat of *T. gerstaeckeri* show a shift to northern areas in USA, with projected suitable habitat in Michigan and in New York (Fig 1B-E). However, distributional shifts northward showed marked differences in habitat suitability between different climate change models and scenarios. For example, CCCMA-A2A and CCCMA-B2A models showed wide regions of unsuitable habitat between extant distributions and future northward shifts (Fig 1B–C). Conversely, CSIRO-A2A and CSIRO-ABA models showed contiguous suitable habitat between extant distribution and future northward shifts (Fig 1D–E). No shifts were observed between extant and future distributions with HADCM3_A2A and HADCM3_B2A models (Fig 1F–G)

Increases in future suitable habitat can be also observed for *T. sanguisuga* through the northeast and northwest of the USA. In all models, north-east shifts showed contiguous habitat suitability. This was not the case for future northwest shifts, where regions of unsuitable habitat were observed between extant and future shifts, except for the CCCMA-A2A model (Fig 2B). In just one model, CCCMA-A2A, the suitable habitat for this vector extended to Florida (Fig 2B). For this species, a shift of suitable habitat to South Texas (Lower Rio Grande Valley) and North Mexico in the State of Tamaulipas is observed using the HADCM3 model for both A2A and B2A (Fig. 2F–G) scenarios of the IPCC, while the CCCMA and CSIRO models (Fig. 2B–E) showing lower suitability habitat compared with the model of present distribution for this region (South Texas-northern Mexico) (Fig. 2A)

For both triatomine species, the variable that contributed the most to the distribution of the species was annual mean temperature (Figs. 1-H and 2-H). The minimum training presence threshold value for *T. gerstaeckeri* was 0.017 and for *T. sanguisuga* 0.068. For *T. gerstaeckeri*, the 0.5 threshold predicted loss on suitable habitat in 2050 compared with the minimum presence threshold for climatic change scenarios, A2A and B2A, and the three general circulation models (CCCMA, CSIRO, and HADCM3) (Table 3). For *T. sanguisuga*, both thresholds predicted an expansion of the suitable habitat by 2050 (Table 3).

**Table 3:** Percentage of change in suitable habitat for *Triotoma gerstaeckeri* and *T. sanguisuga* comparing present and future (year 2050) projections.

Minimum presence threshold	Species	Present km^2^	Scenario A2			Scenario B2		
			CCCMA	CSIRO	HADLEY	CCCMA	CSIRO	HADLEY
	*Triatoma gerstaeckeri*	1903784	63.21	110.89	7.18	87.43	70.58	117.90
	*Triatoma sanguisuga*	2628902	91.18	56.30	61.17	88.64	52.27	40.72
**0.5 threshold**								
	*Triatoma gerstaeckeri*	185879	−94.52	−34.37	−68.36	−55.55	−45.91	−64.05
	*Triatoma sanguisuga*	369908	45.63	113.85	49.49	120.82	72.84	23.85

## Discussion

For both species, *Triatoma gerstaeckeri* and *T. sanguisuga*, our SDMs predicted that there may be range shifts as result of climate change. Species distribution models for *T. gerstaeckeri* [30] and other triatomine species of North America [31] have been developed previously to this paper, but these models were constructed with a coarser spatial resolution (e.g. >1 km^2^). The influence of climatic change has been previously addressed by other authors with a consideration of three triatomine species (*T. lecticularia*, *T. protacta*, and *T. sanguisuga*) [30]. However, our analysis is the first attempt to model future distribution of suitable habitat for *Triatoma gersteckeri* and *T. sanguisuga* performed with the knowledge that all specimens were professionally identified and all locations for the species were explicitly reviewed for accuracy in their geography and method of recording (GPS coordinates with >1m error) and with a finer spatial resolution (1 km^2^). In addition, the cross validation and the low standard deviations in the model evaluations show no sampling biases attributed to the heterogeneity in the source of data and insect collection protocols. That is, models were neither strengthened nor weakened by the inclusion or exclusion of localities chosen based on this information.

Our results support [32] the conclusion that an increase in temperature is correlated with a potential increase of Chagas disease risk, defined as shifts in suitable habitat of *T. gerstaeckeri* and *T. sanguisuga* in the United States. Future distribution models showed marked differences for both triatomine species with important consequences for predicting Chagas disease risk. Overall, future distributions for *T. gerstaeckeri* showed wide discontinuous regions of suitable habitat between extant distributions and north-east shifts in the US. Thus, future north-east shifts of *T. gerstaeckeri* will depend heavily on natural abilities of this triatomine to disperse across wide regions of unsuitable habitat or to be transported by humans, except for CSIRO-A2A model showing more contiguous suitable habitat (Fig 1D). Two models, HADCM3_A2A, and HADCM3_B2A, did not predict northward shifts of this triatomine to Michigan and New York (Fig 1F–G).

Predicted north-east shifts of *T. sanguisuga* suggest contiguous suitable habitat, facilitating potential dispersal of this species to Michigan and New York (Fig 2A–G). Thus, *T. sanguisuga* is the target species most likely to be a threat of spreading Chagas disease in the north-eastern US, although this species is not considered an efficient vector for transmitting the parasite to humans [33]. Conversely, a different Chagas disease risk resulted for future shifts in northwest US. For both triatomine species, north-west shifts included wide areas of discontinuous suitable habitat between extant and future distributions (excluding *T. sanguisuga* in the CCCMA-A2A model). Thus, future shifts necessarily require high dispersal abilities for both triatomine species to represent a Chagas disease risk in north-west US. Other similar studies have identified important future shifts in north-east United States for other vector-borne diseases such as leishmaniasis [5]. Future distributional shifts of vector species can help to forecast expected number of human individuals potentially exposed to infectious diseases under climate change scenarios.

In addition to climate, several other factors not considered in this analysis could influence the distribution of the insects both under present circumstances and future ones. These factors can be biological (i.e., species interactions: competition, parasitism and trophic interactions), historical (e.g., barriers and speciation process), geographic (capabilities of dispersion, accessible regions for dispersal, evolutionary capacity of species' populations to adapt to new conditions), and/or anthropogenic[34], [35]. However, climatic variables (abiotic factors) are frequently used to estimate species' distributions [36], [37] since climate can limit distributions directly by affecting growth or survival (e.g., lower and upper lethal temperatures), and indirectly via interacting species (e.g., food sources, pathogens, competitors, or predators). Additionally, mechanism-based analysis have shown that temperature might have a strong influence on the behavior of triatomine species [20], [21], increasing their feeding rate when temperature increases and humidity is low, or by developing shorter life cycles and higher population densities [20]. High temperatures can also speed up the development of *T. cruzi* in vectors [22]. Therefore, as seen in our results, changes in temperature and precipitation based on the different climate change scenarios and general circulatory models can positively influence the spread of triatomine species to non-original distribution in North America.

Any study that attempts to evaluate the risk for Chagas disease should focus on the ecology and biogeography of triatomine vectors and reservoir species (e.g., woodrats), as well as the incidence of the parasite that causes the disease, *Trypanosoma cruzi* [7]. There is currently research to develop a vaccine for Chagas disease [9], but this is not available yet and drug treatments have limited efficacy. Chagas disease is controlled by using insecticides and improvements in housing, but such publicly organized programs do not exist in the United States, partly due to lack of information regarding human cases, vector-parasite incidence, and reservoirs of the disease. Studies that can provide baseline data for addressing these critical concerns should combine field work, molecular analysis (e.g., examining blood meals of triatomines) and ecological modeling techniques to assess the potential for Chagas disease at a fine-geographic scale (e.g., areas at most risk for Chagas disease; see [38]) are encouraged. Findings from that work can be used to advise health program managers in their efforts to control or prevent transmission of Chagas disease effectively and provide a cost-effective method of predicting locations of high transmission risk of this disease, particularly in light of the economic burden that Chagas disease might represent (similar or higher than other diseases such as rotavirus, cervical cancer, or Lyme disease [39]).

### Concluding remarks

Although we acknowledge several important shortcomings discussed below, our study emphasizes one issue that has not been previously considered: the importance of climate change in the transmission of *T. cruzi*.

The transmission of *T. cruzi* includes several vectors and hosts in domestic, peri-domestic, and sylvatic cycles. *Trypanosoma. cruzi* has three infective forms capable of infecting its host, and currently 6 DTUs (discrete typing units) are recognized in the taxon. These DTUs establish with mammalian hosts peculiar interactions in distinct time-space scales. Thus, the transmission of *T. cruzi* is a complex system for its non-linearity, unpredictability and also for being multivariable.

Ideally, the potential distribution of most hosts should be included in the modeling exercises. We know relatively little about which mammal species are confirmed hosts of *T. cruzi*. To include simply a large list of mammals into the modeling approach without the certainty of being confirmed hosts of this parasite will add confusion into our understanding of this crucial biotic interaction. More studies are needed to produce a comprehensive list of confirmed hosts for *T. cruzi* as well as time-space scales for the operative interactions of hosts, vectors, and parasites. Novel modeling techniques developed to provide a predictive list of potential hosts for other emerging diseases, such as leishmaniasis [40], can be applied for *T. cruzi*.

Landscape and ecotypic scenarios under climate change are also needed to refine distribution shifts of species at finer spatial scales. This information should be associated with data on the salient features of landscape diversity, roles of extant members of regional mammalian faunas, local cultural, social and economic diversity, as well as the land use practices. This information will provide a more comprehensive understanding of the complexity in the transmission of *T. cruzi*.

## Supporting Information

Table S1 **Geographic localities for** ***Triatoma gerstaeckeri*** **.** Only post-1980 records with an estimated error <1 km were used; these choices ensured compatibility between the resolution of the occurrence data and the spatial and temporal resolution of the environmental layers.
(DOCX)

Table S2 **Geographic localities for** ***Triatoma sanguisuga*** **.** Only post-1980 records with an estimated error <1 km were used; these choices ensured compatibility between the resolution of the occurrence data and the spatial and temporal resolution of the environmental layers.
(DOCX)
