# Supplementary material for: Projected Future Distributions of Vectors of Trypanosoma cruzi in North America under Climate Change Scenarios
Source: PLoS Negl Trop Dis. 2014 May 15;8(5):e2818. doi: 10.1371/journal.pntd.0002818 (PMC4022587; doi:10.1371/journal.pntd.0002818)
Supplement: Table S1 — Geographic localities for Triatoma gerstaeckeri . Only post-1980 records with an estimated error <1 km were used; these choices ensured compatibility between the resolution of the occurrence data and the spatial and temporal resolution of the environmental layers. (DOCX) [file pntd.0002818.s001.docx]

Supplemental Table 1. Geographic localities for *Triatoma gerstaeckeri*. Only post-1980 records with an estimated error <1km were used; these choices ensured compatibility between the resolution of the occurrence data and the spatial and temporal resolution of the environmental layers.

| Species | | Latitude | Longitude | Year of collection | |
| --- | --- | --- | --- | --- | --- |
| *Triatoma gerstaeckeri* | 29.5085 | -98.4568 | 1981 |  |  |
|  | 27.9540 | -97.7720 | 1981 |  |  |
|  | 32.8330 | -97.9850 | 1982 |  |  |
|  | 25.6890 | -100.1380 | 1984 |  |  |
|  | 34.5510 | -102.3110 | 1985 |  |  |
|  | 30.4727 | -99.7787 | 1986 |  |  |
|  | 29.6395 | -98.4242 | 1987 |  |  |
|  | 29.6069 | -100.4401 | 1987 |  |  |
|  | 29.6051 | -100.4433 | 1988 |  |  |
|  | 24.6760 | -103.3460 | 1988 |  |  |
|  | 30.2842 | -97.7783 | 1988 |  |  |
|  | 30.4727 | -99.7787 | 1988 |  |  |
|  | 30.2610 | -97.8181 | 1990 |  |  |
|  | 30.6770 | -97.7216 | 1990 |  |  |
|  | 30.6037 | -97.7246 | 1990 |  |  |
|  | 30.6543 | -98.0773 | 1990 |  |  |
|  | 30.6859 | -97.7665 | 1991 |  |  |
|  | 30.4083 | -97.8551 | 1991 |  |  |
|  | 31.3503 | -97.7922 | 1992 |  |  |
|  | 29.4538 | -98.7870 | 1993 |  |  |
|  | 30.2842 | -97.7783 | 1993 |  |  |
|  | 30.3836 | -97.7773 | 1993 |  |  |
|  | 29.5559 | -98.7514 | 1994 |  |  |
|  | 30.7113 | -97.7172 | 1994 |  |  |
|  | 30.7387 | -97.7410 | 1994 |  |  |
|  | 30.7413 | -97.7444 | 1994 |  |  |
|  | 30.5430 | -97.7097 | 1994 |  |  |
|  | 31.1505 | -97.6530 | 1995 |  |  |
|  | 29.4956 | -99.7143 | 1999 |  |  |
|  | 29.3840 | -94.9020 | 1999 |  |  |
|  | 30.2842 | -97.7783 | 1999 |  |  |
|  | 28.8853 | -100.9941 | 2000 |  |  |
|  | 29.2700 | -103.3000 | 2002 |  |  |
|  | 30.2860 | -97.3210 | 2002 |  |  |
|  | 29.8850 | -100.9940 | 2003 |  |  |
|  | 28.3278 | -99.4110 | 2003 |  |  |
|  | 28.3016 | -99.3980 | 2003 |  |  |
|  | 28.3015 | -99.3979 | 2003 |  |  |
|  | 31.1853 | -97.5357 | 2003 |  |  |
|  | 30.4500 | -101.7310 | 2003 |  |  |
|  | 30.2860 | -97.3210 | 2004 |  |  |
|  | 30.4665 | -101.8008 | 2005 |  |  |
|  | 29.2693 | -103.3009 | 2006 |  |  |
|  | 31.6573 | -98.4629 | 2006 |  |  |
|  | 30.3907 | -97.8595 | 2006 |  |  |
|  | 30.6510 | -98.5849 | 2006 |  |  |
|  | 29.4596 | -100.9951 | 2007 |  |  |
|  | 29.7246 | -100.6443 | 2007 |  |  |
|  | 31.0556 | -98.5012 | 2008 |  |  |
|  | 29.5681 | -99.2500 | 2009 |  |  |
|  | 30.2840 | -97.7790 | 2009 |  |  |
|  | 30.2500 | -97.5680 | 2009 |  |  |
|  | 30.1860 | -97.8730 | 2009 |  |  |
|  | 29.8615 | -97.9984 | 2009 |  |  |
|  | 26.5389 | -100.4674 | 2009 |  |  |
|  | 29.3140 | -97.7580 | 2009 |  |  |
|  | 31.1687 | -97.6598 | 2009 |  |  |
|  | 30.7112 | -97.7238 | 2009 |  |  |
|  | 31.2042 | -97.5237 | 2009 |  |  |
|  | 30.3873 | -97.7116 | 2009 |  |  |
|  | 30.6297 | -98.1086 | 2009 |  |  |
|  | 30.5990 | -103.9290 | 2009 |  |  |
|  | 29.5680 | -99.2500 | 2010 |  |  |
|  | 29.3490 | -99.7620 | 2010 |  |  |
|  | 30.0890 | -97.8990 | 2010 |  |  |
|  | 30.0886 | -97.8989 | 2010 |  |  |
|  | 29.8850 | -103.2210 | 2010 |  |  |
|  | 28.9030 | -102.5470 | 2010 |  |  |
|  | 29.3490 | -99.7660 | 2010 |  |  |
|  | 29.3490 | -99.7660 | 2010 |  |  |
|  | 29.3140 | -97.7580 | 2010 |  |  |
|  | 30.8500 | -98.1330 | 2010 |  |  |
|  | 32.7887 | -98.4891 | 2010 |  |  |
|  | 29.3614 | -97.3460 | 2011 |  |  |
|  | 29.7768 | -99.5224 | 2011 |  |  |
|  | 30.1372 | -98.0306 | 2011 |  |  |
|  | 26.5269 | -98.0751 | 2011 |  |  |
|  | 26.4084 | -98.4588 | 2011 |  |  |
|  | 26.4080 | -98.4590 | 2011 |  |  |
|  | 26.3988 | -98.3445 | 2011 |  |  |
|  | 26.3129 | -97.9961 | 2011 |  |  |
|  | 26.5713 | -98.8894 | 2011 |  |  |
|  | 26.0975 | -98.2548 | 2011 |  |  |
|  | 26.0738 | -97.2272 | 2011 |  |  |
|  | 24.7931 | -99.5418 | 2011 |  |  |
|  | 29.3488 | -99.7659 | 2011 |  |  |
|  | 26.5309 | -98.0703 | 2011 |  |  |
|  | 29.1850 | -99.1793 | 2011 |  |  |
|  | 30.8500 | -98.1336 | 2011 |  |  |
|  | 33.5436 | -101.8914 | 2011 |  |  |
|  | 30.4745 | -97.8579 | 2011 |  |  |
|  | 29.6402 | -98.6584 | 2012 |  |  |
|  | 29.5867 | -99.2506 | 2012 |  |  |
|  | 29.7856 | -97.8759 | 2012 |  |  |
|  | 29.5783 | -99.2506 | 2012 |  |  |
|  | 30.1021 | -97.5702 | 2012 |  |  |
|  | 29.7856 | -97.8757 | 2012 |  |  |
|  | 30.1466 | -97.9563 | 2012 |  |  |
|  | 26.1813 | -98.3506 | 2012 |  |  |
|  | 26.1813 | -98.3506 | 2012 |  |  |
|  | 26.1812 | -98.3506 | 2012 |  |  |
|  | 26.1270 | -97.6237 | 2012 |  |  |
|  | 26.1813 | -98.3506 | 2012 |  |  |
|  | 28.5860 | -99.1773 | 2012 |  |  |
|  | 30.7276 | -97.9272 | 2012 |  |  |
