# Supplementary material for: Projected Future Distributions of Vectors of Trypanosoma cruzi in North America under Climate Change Scenarios
Source: PLoS Negl Trop Dis. 2014 May 15;8(5):e2818. doi: 10.1371/journal.pntd.0002818 (PMC4022587; doi:10.1371/journal.pntd.0002818)
Supplement: Table S2 — Geographic localities for Triatoma sanguisuga . Only post-1980 records with an estimated error <1 km were used; these choices ensured compatibility between the resolution of the occurrence data and the spatial and temporal resolution of the environmental layers. (DOCX) [file pntd.0002818.s002.docx]

Supplemental Table 2.Geographic localities for *Triatoma sanguisuga*. Only post-1980 records with an estimated error <1km were used; these choices ensured compatibility between the resolution of the occurrence data and the spatial and temporal resolution of the environmental layers.

| *Species* | Latitude | Longitude | Year of collection |
| --- | --- | --- | --- |
| *Triatomasanguisuga* | 30.2842 | -97.7783 | 1983 |
|  | 30.4727 | -99.7787 | 1986 |
|  | 37.3884 | -91.2653 | 1989 |
|  | 38.2440 | -82.4370 | 1989 |
|  | 34.5153 | -111.7625 | 1999 |
|  | 30.2842 | -97.7783 | 1999 |
|  | 30.5879 | -96.2532 | 2001 |
|  | 30.5883 | -96.2533 | 2001 |
|  | 30.5722 | -97.7535 | 2002 |
|  | 30.7894 | -98.3550 | 2003 |
|  | 30.5879 | -96.2532 | 2003 |
|  | 33.5534 | -101.8791 | 2003 |
|  | 30.5879 | -96.2532 | 2004 |
|  | 33.0240 | -84.7160 | 2004 |
|  | 33.5436 | -101.8914 | 2006 |
|  | 30.2170 | -97.3750 | 2009 |
|  | 30.1830 | -97.4200 | 2009 |
|  | 30.1860 | -97.8730 | 2009 |
|  | 32.8830 | -98.4530 | 2010 |
|  | 30.5250 | -97.6470 | 2010 |
|  | 30.2354 | -98.1858 | 2011 |
|  | 30.2170 | -97.3750 | 2011 |
|  | 30.2167 | -97.4251 | 2011 |
|  | 30.2167 | -97.4251 | 2011 |
|  | 29.3614 | -97.3460 | 2011 |
|  | 29.1850 | -99.1793 | 2011 |
|  | 26.5309 | -98.0703 | 2011 |
|  | 29.6402 | -98.6584 | 2012 |
